# Stone Formation Due to Migration of Hemostatic Clip After Robot-Assisted Laparoscopic Radical Prostatectomy: A Late and Rare Presentation

**DOI:** 10.7759/cureus.30922

**Published:** 2022-10-31

**Authors:** Shameer Deen, Omer Rehman, Rahul Lunawat, Ali Tasleem

**Affiliations:** 1 Urology, Princess Royal University Hospital, London, GBR

**Keywords:** uroncology and stone disease, ralp, robotic prostatectomy, prostate cancer, hemostatic clip

## Abstract

A very rare complication of robot-assisted laparoscopic radical prostatectomy (RALP) is bladder stone formation from a hemostatic clip as a nidus. A 70-year-old man presented 11 years after a RALP with recurrent UTI, worsening lower urinary tract symptoms, and visible haematuria. A flexible cystoscopy revealed a large 3.5 cm x 1.5 cm stone at the bladder neck. Computed Tomography (CT) Urogram revealed normal kidneys with no hydronephrosis and a bladder stone. The patient underwent a cystolithotripsy as a day case which was performed by use of Swiss LithoClast® Trilogy lithotripter, which delivered controlled ultrasonic and ballistic energy and simultaneous suction through a single probe. Hemostatic clips were incidentally identified during the procedure and were successfully removed. The patient was discharged on the same day and made an excellent recovery. Hence, migration of clips is a rare occurrence, and good intraoperative techniques should avoid such complications to occur.

## Introduction

Prostate cancer is a commonly diagnosed malignant tumor in men [1]. The disease is risk-stratified, and treatment is tailored. Surgical excision of the prostate is usually reserved for intermediate-high or high-risk organ-confined disease or if it's the patient’s choice. Minimal access surgery has proved its benefit with small incisions, lower risk of bleeding and postoperative complications with good recovery periods, and shorter stays [2]. Radical prostatectomy, either retropubic laparoscopic or robot-assisted laparoscopic are standard of choice for surgical removal of the prostate, and the use of Hem-o-Lok, a nonabsorbable polymer clip has been the standard surgical practice since 1999 to stop intraoperative bleeding and aids in closing tissue structures [3]. Bladder stones forming over migrated hemostatic clips after RALP is very rare, with a variable time of presentation [4]. We report the case of a patient developing a bladder stone over a migrated hemostatic clip 11 years after RALP.

## Case presentation

A 70-year-old man was reviewed on the two-week wait pathway for visible haematuria and worsening lower urinary tract symptoms (LUTS). He was diagnosed with high-risk Gleason 3+4=7 organ-confined (T3aN0M0) adenocarcinoma of the prostate in 2011 and successfully underwent a robot-assisted laparoscopic radical prostatectomy with no post-operative complications. He remained well for almost 11 years with an undetectable PSA. He was recently referred by the general practitioner with a three-month history of worsening LUTS and new onset visible haematuria. When questioned about his symptoms, he described increased frequency, incomplete bladder emptying, urgency, and dysuria.

**Figure 1:**
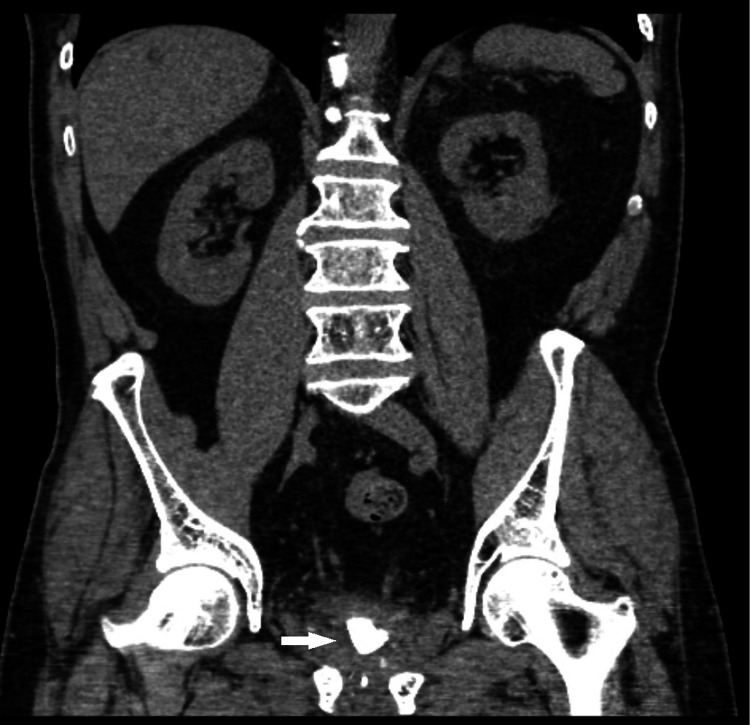
CT Abdomen coronal view depicting large bladder stone at level of bladder neck

**Figure 2:**
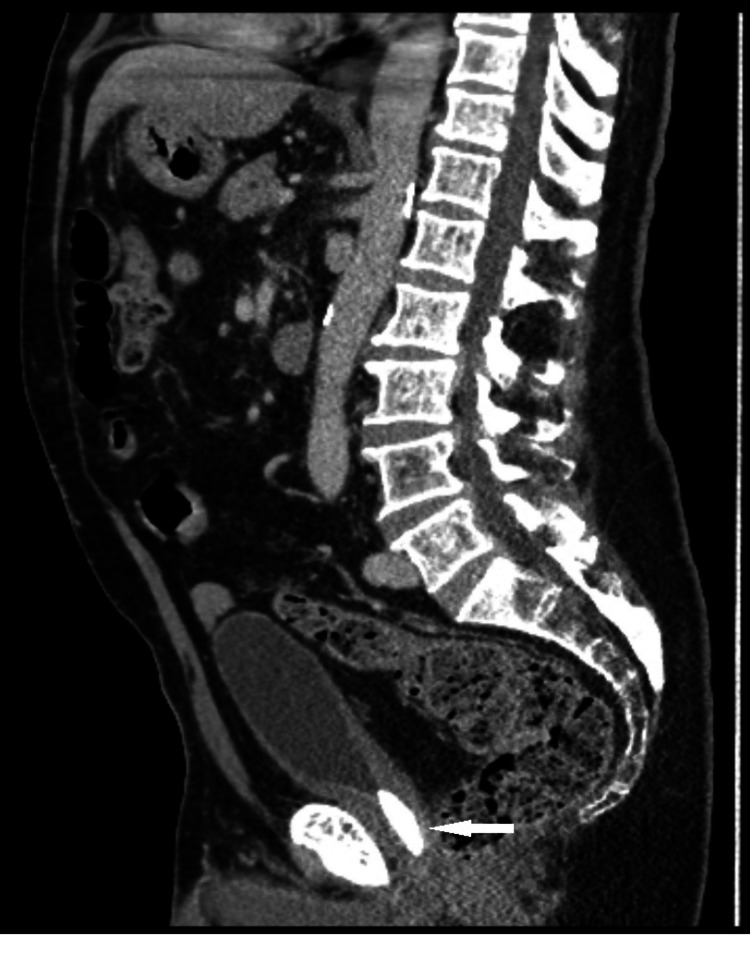
CT Abdomen saggittal view depicting large bladder stone at level of bladder neck

On examination, the patient had an unremarkable cardiorespiratory system. Abdominal examination revealed a soft, non-tender abdomen. A bladder scan revealed significant residual urine of 150ml, and his serum prostate-specific antigen (PSA) was untraceable at <0.01. Flexible cystoscopy revealed a normal anterior urethra, the sphincter was seen, and immediately proximal to this was a large bladder stone with difficult negotiation of the flexible cystoscope. Both ureteric orifices were identified after the washout of debris in the bladder, and no cancer was identified in the bladder. A digital rectal exam (DRE) revealed no prostate or masses. Abdominal ultrasonographic examination revealed a large bladder stone at the bladder neck and normal kidneys. A CT urogram as part of the evaluation of visible haematuria revealed normal kidneys, simple renal cysts, no hydronephrosis or renal lesions, and a 3.5 cm stone at the level of the bladder neck (Figure 1) and (Figure 2).

**Figure 3:**
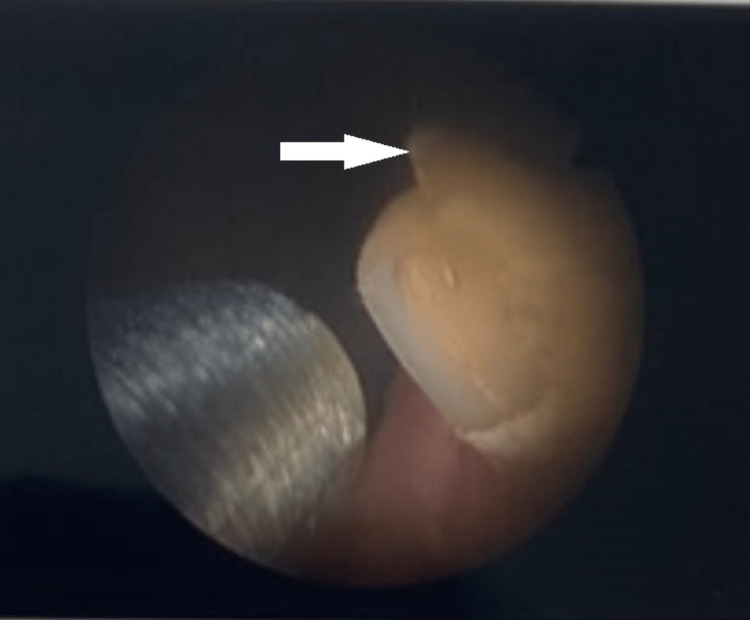
Lithoclast fragmentation of a yellow-colored bladder stone

**Figure 4:**
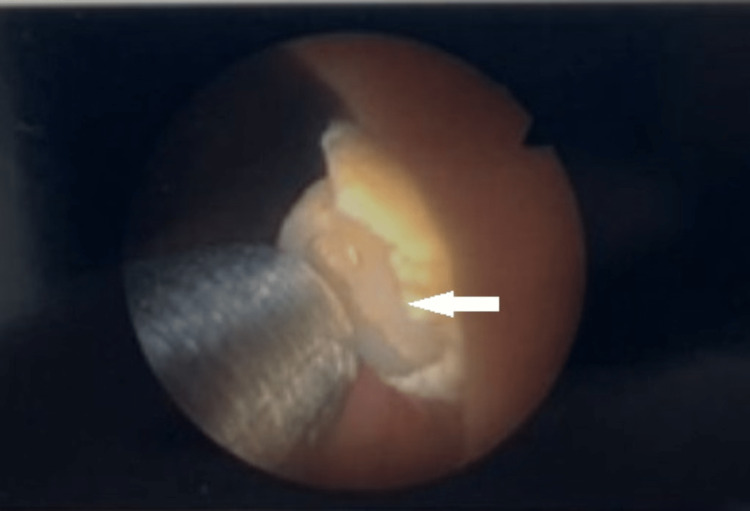
Fragmentation revealed a hemostatic clip as a nidus

**Figure 5:**
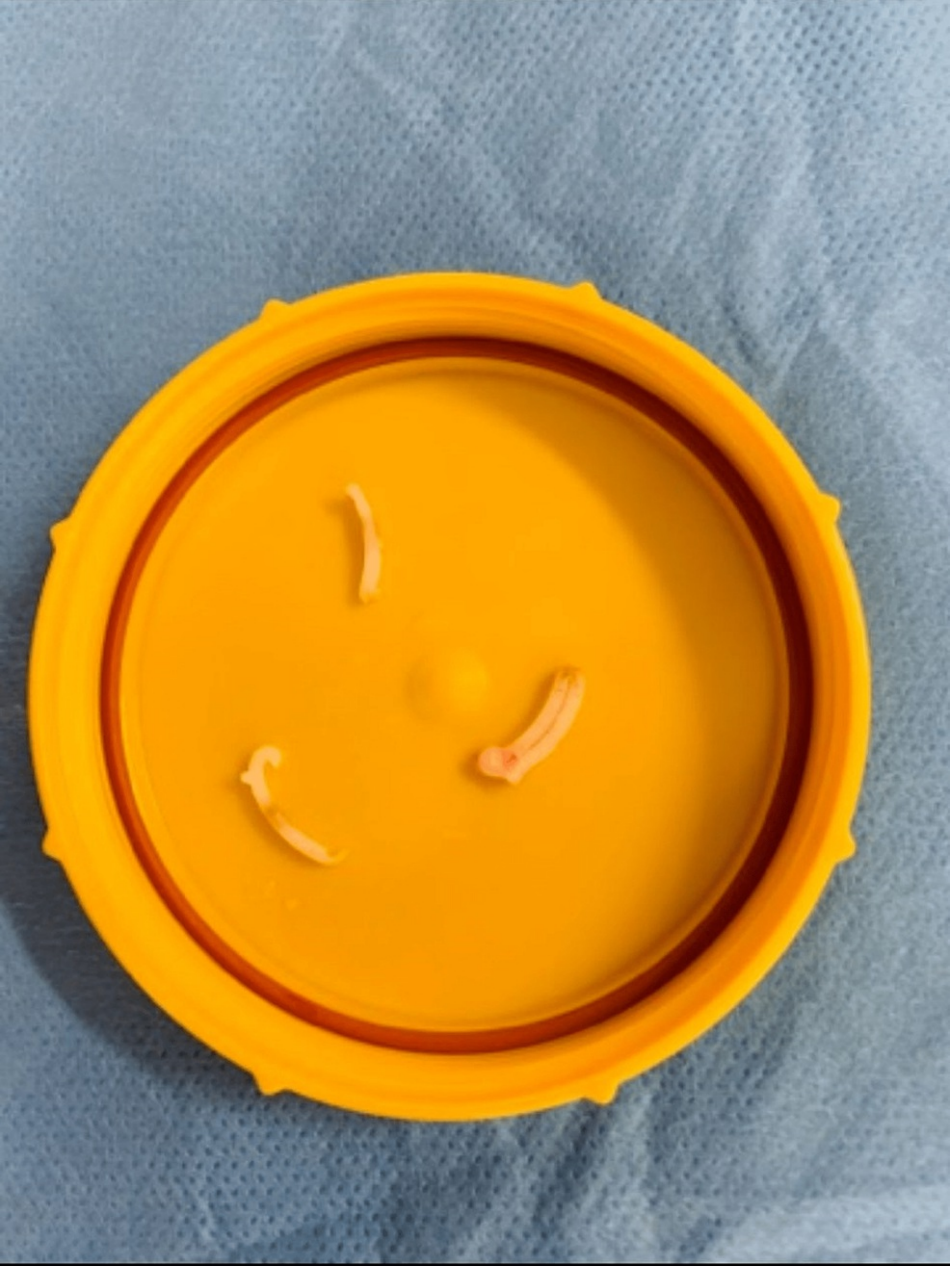
Hemostatic clips removed at completion of the procedure

The patient was electively admitted to the day surgery unit and underwent stone fragmentation with a Swiss LithoClast® Trilogy lithotripter to a yellow-colored stone (Figure 3) at the level of the bladder neck, as the stone gradually disintegrated, a hemostatic clip was identified as the nidus (Figure 4). The stone was completely fragmented, and the clips were recovered (Figure 5). The patient was discharged on the same day without a catheter and was taken off the two-week-wait haematuria pathway. He was later reviewed by the urology outpatients, and was informed of the complete resolution of his symptoms, and no further haematuria occurred.

## Discussion

Hem-o-Lok® clips (HOLCs) (Weck® Surgical Instruments, Teleflex Medical, Durham, NC) are widely used for controlling the lateral pedicles in laparoscopic radical prostatectomy [5]. These are available in medium, medium-large, large, and extra-large sizes. The mechanism of clip migration can be either due to improper placement, postsurgical inflammation and its resolution, true migration to the bladder neck due to scar formation and/or fibrosis, or direct erosion with time. Migrations are classified into three types: Type I migration resulted in obstructive lower urinary tract symptoms 2-8 months after prostatectomy, whereas Type II migration led to stone formation, gross haematuria, or bladder spasm; in Type III migration, patients had spontaneous expulsion of the HOLC weeks after surgery [5].

The duration of the symptoms manifesting is variable, with some cases presenting within less than 1-2 years of the surgery [4]. Our patient returned after a hiatus of 11 years, which is much later than the usual time of presentation reported in the literature. The migrated hemostatic clip likely led to a gradual build-up of bladder stone. With improvement in the technique of the surgery, this complication has not been reported again in our network.

The literature review identified approximately nine cases. Tugcu V et al. [6], in 2009, where two patients presented with migration; however, we were unable to ascertain if these cases were done with the assistance of a robot. The recommendation to use such devices with caution around vesicourethral anastomosis is valid. Aoki T et al. [7], in 2016 reported a case report of a 54-year-old patient who underwent a robot-assisted laparoscopic radical prostatectomy in June 2014 and presented three months later with lower urinary tract symptoms and haematuria and a stone with Hem-o-lock as nidus was removed and the patient made a good recovery.

George A. TuriniIII et al. [8] in September 2016 presented the largest case series and systematic review of the literature. Five hundred and seventy patients were included in the study and the incidence of migration of hemostatic clips with resultant bladder stones in eight patients (1.4%). Another case series and review were conducted by Jun Seok Yi et al. [9], where 641 patients underwent radical prostatectomy, and the authors identified hemostatic clip-related complications in six patients of which two patients had bladder stones, both underwent open retropubic prostatectomies, it was aptly summarized that, due to incidence of bladder neck contractures after RALP (1.3%), migration of Hem-o-Lok clips should be suspected when patients present with lower urinary tract symptoms.

## Conclusions

Migration of hemostatic clip and bladder stone formation after robotic laparoscopic radical prostatectomy can present as irritable lower urinary tract symptoms, visible haematuria, and urinary tract infections irrelevant to time or duration after surgery. However, such occurrences are also rare. Good intraoperative technique and care should be taken to avoid such complications.
